# After a century of nisin research - where are we now?

**DOI:** 10.1093/femsre/fuad023

**Published:** 2023-05-11

**Authors:** Des Field, Miguel Fernandez de Ullivarri, R Paul Ross, Colin Hill

**Affiliations:** APC Microbiome Ireland, University College Cork,Western Road, Cork T12 YN60, Ireland; School of Microbiology, University College Cork, College Road, Cork T12 YT20, Ireland; APC Microbiome Ireland, University College Cork,Western Road, Cork T12 YN60, Ireland; APC Microbiome Ireland, University College Cork,Western Road, Cork T12 YN60, Ireland; School of Microbiology, University College Cork, College Road, Cork T12 YT20, Ireland; APC Microbiome Ireland, University College Cork,Western Road, Cork T12 YN60, Ireland; School of Microbiology, University College Cork, College Road, Cork T12 YT20, Ireland

**Keywords:** antimicrobial, lantibiotic, microbiome, nisin, bioengineering, antibiotic resistance

## Abstract

It is almost a century since nisin was discovered in fermented milk cultures, coincidentally in the same year that penicillin was first described. Over the last 100 years this small, highly modified pentacyclic peptide has not only found success in the food industry as a preservative but has also served as the paradigm for our understanding of the genetic organization, expression, and regulation of genes involved in lantibiotic biosynthesis—one of the few cases of extensive post-translation modification in prokaryotes. Recent developments in understanding the complex biosynthesis of nisin have shed light on the cellular location of the modification and transport machinery and the co-ordinated series of spatio-temporal events required to produce active nisin and provide resistance and immunity. The continued unearthing of new natural variants from within human and animal gastrointestinal tracts has sparked interest in the potential application of nisin to influence the microbiome, given the growing recognition of the role the gastrointestinal microbiota plays in health and disease. Moreover, interdisciplinary approaches have taken advantage of biotechnological advancements to bioengineer nisin to produce novel variants and expand nisin functionality for applications in the biomedical field. This review will discuss the latest progress in these aspects of nisin research.

## Teaser

The highly post-translationally modified peptide nisin has been studied over the course of the last 100 years, and although it has been employed successfully as a food preservative, its potent activity against multidrug resistant microbes, long safety record, lack of any significant resistance development, and amenability to bioengineering approaches to improve its antimicrobial and physicochemical properties has meant that the focus on nisin-related research is shifting from food preservation towards therapeutic use for the treatment of bacterial infections.

## Introduction

Nisin is one of the oldest known antimicrobial compounds. In 1928, the same year that Alexander Fleming discovered penicillin, Rogers and Whittier (Rogers 1928) noted that certain lactic streptococci (then called Group N *Streptococcus*) were inhibitory to starter cheese cultures. Almost 20 years following its discovery, this inhibitory peptide was named nisin, or ‘group *N Streptococcus I*nhibitory *S*ubstance’, the suffix ‘-in’ implying antibacterial properties (Hirsch and Mattick 1949). Although initially proposed for use as an antibiotic based on its activity against veterinary and clinical pathogens including *Mycobacterium tuberculosis*, it was deemed unsuitable due to low aqueous solubility and poor stability at physiological pH (Hurst 1981). However, in the 1950s the potency of nisin as a food preservative was realized based on its success in preventing spoilage of processed cheese by clostridia (Delves-Broughton et al. 1996). Since then, this highly modified peptide has gained approval by regulators in over 80 countries, including the Food and Drug Administration (FDA) and the European Food Safety Authority (EFSA) (Chikindas et al. 2018). Nisin has been used in a wide assortment of foods including dairy and dairy desserts, canned goods, processed meats, fish, fruit juices, and beverages (Gharsallaoui et al. 2016, Todorov et al. 2022). Nisin exhibits potent activity against Gram-positive bacteria including *Bacillus cereus, Listeria monocytogenes*, enterococci, staphylococci, and streptococci. Nisin has also been used in limited applications in the veterinary industry to prevent or treat bovine mastitis (Roy et al. 2016). The commercial success of nisin has made it the most investigated bacteriocin (antimicrobial peptides produced by bacteria that can kill other bacteria) in terms of its genetic organization, biosynthesis, mode of action and efforts to broaden its food and potential therapeutic applications. Nisin is also a lanthipeptide, a rapidly expanding subset of the ribosomally synthesized and post-translationally modified peptides (RiPPs) family (Arnison et al. 2013). Their defining feature is the presence of unusual thioether amino acids including lanthionine (Lan) and/or methyllanthionine (MeLan) that are introduced through a series of enzyme-mediated post-translational modifications (Sahl et al. 2008). Currently, lanthipeptides can be classified into five distinct groups based on the characteristics of their lanthipeptide synthetases. For Class I lanthipeptides, of which nisin is the prototypical member, the thioether cross-linked amino acids (Me)Lan are generated by two distinct enzymes: a dehydratase LanB and cyclase LanC (see later section on nisin biosynthesis). However, in Class II–IV lanthipeptides, multifunctional enzymes are employed to form (Me)Lan, namely LanM (Class II), LanKC (Class III), and LanL (Class IV). A recently discovered group of lanthipeptides that are generated via a biosynthetically distinct pathway since the biosynthetic gene clusters (BGCs) do not contain genes encoding well-defined Class I–IV (Me)Lan synthase homologues constitutes the newest class (Class V) (the reader is referred to excellent reviews covering these aspects in more detail (Hegemann and Süssmuth 2020, Montalbán-López et al. 2021, Lee and van der Donk 2022). Lanthipeptides that exhibit antimicrobial activity have been historically termed lantibiotics. Importantly, some lantibiotics, including nisin, exhibit multiple modes of action, which involve interactions with highly conserved cell wall synthesis intermediates as well as the ability to form pores in the bacterial membrane (Sahl and Bierbaum 2008). In the case of nisin, this dual mode of action and demonstrable high potency against multidrug resistant (MDR) bacteria makes the lantibiotic very attractive for potential use as a biotherapeutic in human and animal health related applications (Mathur et al. 2018, van Staden et al. 2021). Indeed, recent advances in genome mining tools and next generation sequencing (NGS) technologies (Biermann et al. 2022) have facilitated the prediction of novel BGCs from a diverse range of bacteria and sources including human and animal microbiomes. Consequently, the number of natural nisin variants has doubled in the last decade, representing a valuable collection of novel peptide structures that exhibit a range of properties and antimicrobial spectra. Moreover, increasing numbers of metagenomic studies are beginning to shed light on the complex interactions between microbes in the human gastrointestinal tract and the role that antimicrobial peptides might play in this context (Garcia-Gutierrez et al. 2019a, Heilbronner et al. 2021). In fact, recent microbiome-based investigations have revealed that commensal nisin producing bacteria can inhibit MDR pathogens and shape niche competition in the gastrointestinal microbiome, offering exciting prospects for the use of nisin in human therapeutic applications.

The key features of nisin biosynthesis have been painstakingly unravelled over decades, including the regulation and functions of the individual biosynthetic proteins and the complex catalytic processes involved in the formation of the Lan and MeLan rings as well as the export of and immunity to the active peptide (de Vos et al. 1995, Lubelski et al. 2008). However, recent reports have offered a fascinating insight into nisin biosynthesis that also advances our fundamental knowledge regarding the cellular localization, spatial configuration, and complex interaction of the post-translational modification and transport machinery as well as a greater perception of producer self-protection (self-immunity) during nisin biosynthesis. Furthermore, the gene-encoded nature of nisin makes the bacteriocin accessible to biosynthetic engineering through site-directed mutagenesis or synthetic chemistry approaches. Elaborate expression systems in conjunction with high-throughput screening strategies have generated vast collections of nisin derivatives that exhibit altered functional properties. This research has advanced our appreciation of structure–activity relationships, modification enzyme–substrate specificity, and immunity mechanisms that can be used as the basis for the rational design of next generation nisin derivatives with possible applications, which range from activity against Gram-negative bacteria to tools for microbiome editing.

## How is nisin biosynthesized?

**Figure 1.**
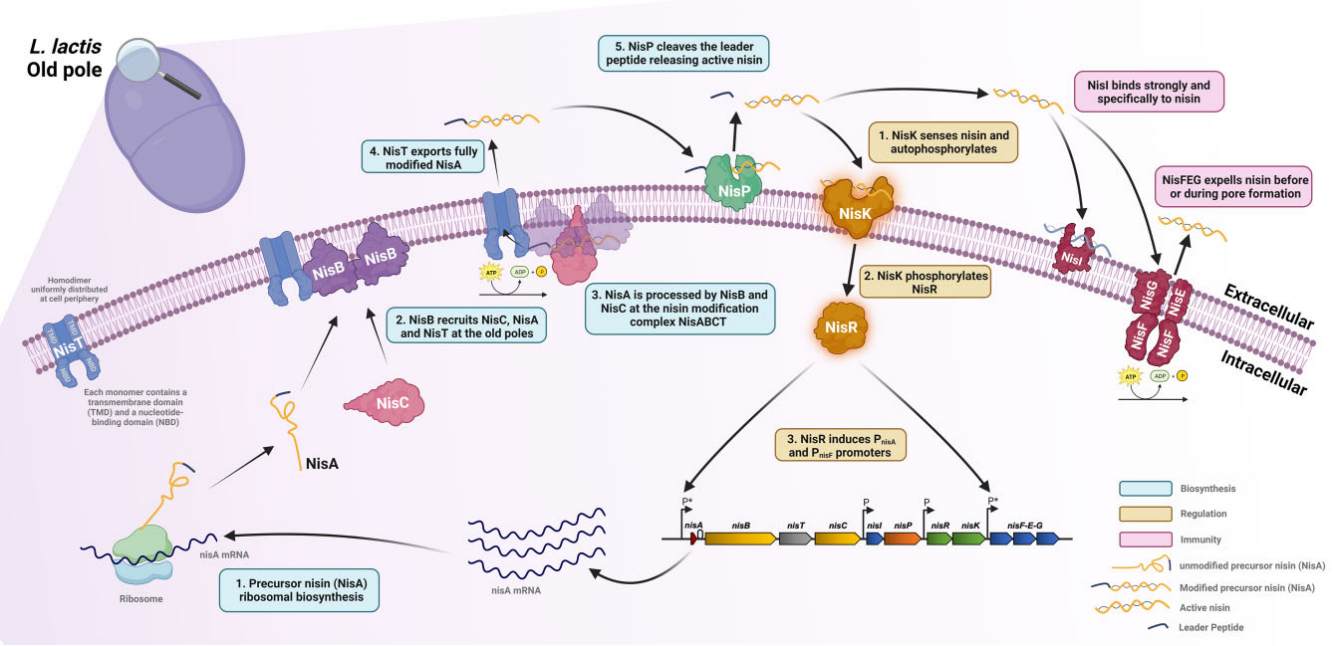
Nisin biosynthesis, regulation, and immunity. Nisin is first synthesized as an unmodified precursor (NisA) consisting of a leader peptide and core peptide. NisA is processed by the dehydratase NisB and cyclase NisC and transported by NisT (NisABCT complex) at the old pole in *L. lactis*. The mature bioactive peptide is released by leader peptide cleavage performed by NisP. Immunity from the active peptide is provided by two distinct immunity systems composed of a lipoprotein NisI and an ABC transporter NisFEG. To ensure a proper balance between production and immunity, nisin expression is regulated *via* a two-component signal TCS composed of a sensor NisK, and response regulator NisR that activate the nisin promoter (P*).

To produce the active peptide, nisin is first ribosomally synthesized as an unmodified 57 amino acid precursor peptide (pre-NisA) consisting of a 23-amino acid N-terminal leader peptide and a 34-amino acid C-terminal core peptide (Fig. 1). The leader peptide serves as a signal sequence for export by NisT and as a recognition motif recognized by the modification enzymes NisB and NisC (Siegers et al. 1996, Kuipers et al. 2006). The first phase in nisin maturation involves the dehydration of selected serine and threonine residues in the core peptide, a process mediated by a dimer of the membrane-associated dehydratase NisB (Fig. 1). This process involves the transfer of glutamate to specific serine and threonine side chains followed by subsequent elimination reactions to generate dehydroalanine and dehydrobutyrine, respectively (Garg et al. 2013). In the next phase, these dehydrated residues are coupled to nearby cysteines via intramolecular addition reactions mediated by the cyclase NisC to form five cyclic bridges composed of a lanthionine (Lan, where an initial serine is linked to a neighbouring cysteine) and 4 methyllanthionine (MeLan, where a threonine is linked to cysteine) rings (Koponen et al. 2002, Li and van der Donk 2007). In the final step, the modified 57 amino acid peptide is exported from the cell via the dedicated ABC-type transporter NisT (Kuipers et al. 2004) and the mature bioactive peptide is released following removal of the leader region by a dedicated serine protease, NisP (Lagedroste et al. 2017, Montalbán-López et al. 2018) (Fig. 1). The producer cell is protected from the now active peptide by two distinct immunity systems composed of a lipoprotein NisI and an ABC transporter NisFEG (Khosa et al. 2016a). To ensure a proper equilibrium between production and immunity, nisin expression is autoregulated by the mature nisin peptide *via* a two-component signal transduction system (TCS) composed of a sensor histidine kinase NisK, and response regulator NisR (Kuipers et al. 1995) (Fig. 1). Binding of mature nisin to NisK stimulates autophosphorylation. The phosphate group is then transferred to activate NisR, which then induces transcription from two of the four promoters in the nisin gene cluster, P*_nisA_ and P*_nisF_ (Fig. 1). Thus, nisin functions as both an antimicrobial peptide and a peptide pheromone that plays an essential role in quorum sensing control of its own biosynthesis (Kleerebezem 2004). A multitude of studies with a particular focus on the individual biosynthetic proteins have been instrumental in elucidating the intricate catalytic processes and stereochemistry involved in formation of the Lan and MeLan residues (For comprehensive reviews see Repka et al. 2017, Montalbán-López et al. 2021). For example, the precise mechanism of the NisB dehydratase action remained elusive due to the inability of researchers to reconstitute NisB activity *in vitro*. This was finally achieved when it was established that NisB required glutamyl-tRNA to bring about the dehydration reaction (Garg et al. 2013). Later studies identified the domains within NisB that catalyse the transfer and subsequent elimination of glutamate from the tRNA to the core peptide to form Dha or Dhb, as well as elucidating the importance of the highly conserved -FNLD- box within the leader peptide for recognition and binding of NisB and NisC to pre-NisA (Abts et al. 2013, Ortega et al. 2015). Furthermore, the requirement of Zn2+ for NisC catalytic activity provided further mechanistic insight into how the cyclase guides the formation of the lanthionine rings (Li and van der Donk 2007). Another important breakthrough revealed the details of the formation of the modification complex which was shown *in vitro* to have a stoichiometry of 2:1:1 (NisB:NisC:pre-NisA) (Reiners et al. 2017a). Additionally, the purification and *in vitro* ATPase activity of NisT was recently demonstrated (Lagedroste et al. 2020). Importantly, numerous reports concerning the organization and cellular location of the nisin modification machinery have alluded to the existence of a membrane-associated multimeric synthetase protein complex consisting of NisB, NisC, and NisT (for a comprehensive review see Lubelski et al. 2008).

However, while these previous studies represent extraordinary advances in their own right, a recent seminal work employed a combination of mutagenesis and fluorescent microscopy to not only confirm the existence of this enzymatic complex, but also to reveal the nature of its assembly and cellular location *in vivo* and the highly co-ordinated series of spatio-temporal events that unfold to produce and export active nisin (Chen et al. 2020). Using an elegantly designed suite of green fluorescent protein (GFP)-labelled NisA fusions as well as sfGFP and mCherry fusions to the N- and/or C-termini of NisB, NisC, and NisT, respectively, it was revealed that pre-NisA binds NisB and NisC to form a complex, i.e. localized to the poles of the *Lactococcus lactis* host (Fig. 1). Further analysis established that NisB and NisC were preferentially located at one pole in single cells, later identified as the ‘old’ pole through time–course experiments. Surprisingly, the transporter NisT was shown to be evenly distributed in the cell periphery and not part of a NisBCT complex as predicted, suggesting that NisT is recruited to the pole localized NisBC complex only when required to transport of the fully modified prepeptide. Indeed, a NisT mutant incapable of secretion and dissociation confirmed this when it was visualized together with NisB and NisC, also at a polar position. Finally, the order of assembly was clarified and revealed that NisB plays a central role in the initial recruitment of the other components (Chen et al. 2020). A short domain (NisB_750–769_) targets dimeric NisB to the pole of the cell. NisC is recruited to form a NisBC complex that interacts with nisin precursor and both enzymes act in succession to perform the dehydration and cyclization reactions and create each lanthionine ring in turn. NisT is drafted to the cell poles from the cytoplasmic membrane only when the modifications are complete and all five rings are in place. The fully modified pre-NisA is then released from NisBC and transferred to NisT. Following export of the fully modified prepeptide, the complex dissociates and NisT again becomes evenly distributed around the cell. It is thought that this polar-localized synthesis and secretion of nisin prevents access of the peptide to its target lipid II, given that peptidoglycan synthesis would be significantly lower at the old pole, thereby limiting the possibility of any self-killing action (Chen et al. 2020). This remarkable insight into the mechanistic details and co-operative spatially controlled actions during nisin biosynthesis could potentially lead to enhanced nisin production and ultimately enable the expression of other rationally designed and novel lanthipeptides.

Indeed, the promiscuity of the nisin enzymatic complex to modify and transport a broad range of substrates attached to the nisin leader sequence, including medically relevant nonlantibiotic peptides (Kluskens et al. 2005, Moll et al. 2010), validates the broader potential applications for the bioengineering of novel compounds other than nisin. A perfect example of this describes the expression of more than 30 novel lantibiotics from almost 60 promising candidate genes identified from genome mining of publicly available prokaryotic sequences (van Heel et al. 2016). The genes were synthesized with the nisin leader peptide sequence and introduced into a nisin expression system. Notably, five modified lantibiotic peptides from a variety of organisms including *Corynebacterium lipophiloflavum* DSM 44291, *Streptococcus agalactiae* ATCC 13813, and *Streptococcus suis* R61, were produced and found to be active against several pathogenic bacteria including vancomycin resistant enterococci (VRE) and methicillin resistant *Staphylococcus aureus* (MRSA) (van Heel et al. 2016). Indeed, efforts to further expand the substrate flexibility of biosynthetic enzymes provides even more tantalizing prospects for novel and bioengineered peptides with unique functionalities. For example, a recent study employed a random mutagenesis approach to generate mutant libraries of the dehydratase NisB (Zhao et al. 2020a). A high throughput selection strategy based on cell surface display of modified and cyclized peptides identified a NisB derivative exhibiting broader substrate flexibility with a capacity to modify substrates normally incompatible with the wild type NisB.

## Nisin resistance and immunity mechanisms

**Figure 2.**
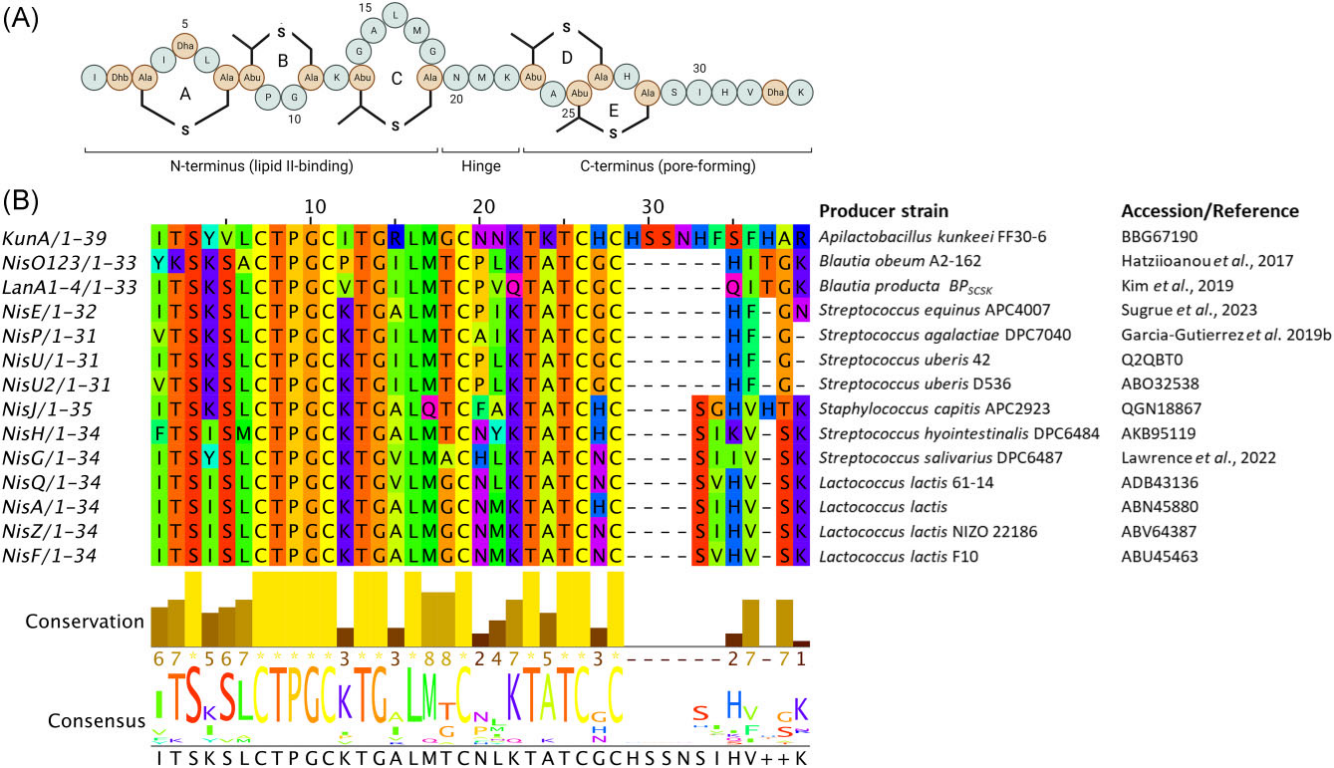
Multiple-sequence alignment of all natural nisin variants characterized to date. Alignment generated from MUSCLE (plotted using http://msa.biojs.net/app/). The total height of the sequence logo at each position reflects the degree of conservation at that position in the alignment, while the height of each letter in that position is proportional to the observed frequency of the corresponding amino acid at that position. A reference is provided where the accession number is not available.

Given the remarkable ability of microbes to adapt to their environments, it is not surprising that persistent bacterial exposure to bacteriocins can lead to resistance development (Kumariya et al. 2019). Resistance to lantibiotics has been noted, with the most frequent mechanisms involving physiological adaptations to the cell-envelope including cell wall thickening, alterations to phospholipid and membrane fatty acid composition, and the overall cell wall charge (The reader is directed to comprehensive reviews describing these mechanisms in greater detail; Bastos et al. 2015, Draper et al. 2015, Barbosa et al. 2021). Over the last decade, bacterial resistance mediated by transporters has gained significant attention due to the high degree of protection these systems provide against cell wall-active drugs, including antimicrobial peptides (Gebhard 2012). Although a wide variety of these integral membrane proteins have been characterized as immunity systems in producing organisms, they have also been identified in the genomes of nonproducing and often pathogenic organisms. To understand how these transporters confer resistance to nisin, it is first necessary to appreciate the mode of action of the peptide. Nisin exerts its antimicrobial action both by pore formation and by inhibition of cell wall synthesis through specific binding to lipid II, an essential precursor in peptidoglycan biosynthesis (Breukink et al. 1999, Wiedemann et al. 2001). This dual functionality is mediated by two structural domains, an N-terminal domain composed of rings A, B, and C, linked to the C-terminal rings (D and E) by a short three residue hinge region (Fig. 2A). Studies have revealed that the N-terminal lanthionine rings form a pyrophosphate cage around the head-group of lipid II, thus inhibiting cell wall synthesis. The interaction primarily occurs via five hydrogen bonds formed between the amide backbone of rings A and B of nisin and the pyrophosphate moiety of lipid II (Hsu et al. 2004). This binding enhances insertion of rings D and E in a transmembrane orientation, facilitated by the flexible hinge, forming a stable pore. Notably, lipid II is also the target of the glyco- and lipoglyco-peptide antibiotics vancomycin, teicoplanin, telavancin, dalbavancin, and oritavancin that serve as first line antibiotics to treat MDR Gram-positive pathogens. This is also true for the depsipeptides ramoplanin and teixobactin, though they bind different segments of the highly conserved lipid II molecule than nisin, which has relevance for both mode of action and mode of resistance (Ulm and Schneider 2016). In the case of nisin, binding to lipid II also facilitates pore formation leading to the rapid efflux of ions and small cytoplasmic compounds from the target organism (Bierbaum and Sahl 2009). Initially, it was believed that the pores consisted of eight nisin and four lipid II molecules (Hasper et al. 2004), though recent evidence suggests the pore complex grows continuously through the recruitment of more and more nisin–lipid II aggregates leading to immense membrane damage (Scherer et al. 2015). The producer strain escapes the killing action of nisin by expressing a set of nisin-specific immunity proteins consisting of a membrane-anchored lipoprotein NisI and a multiprotein ABC-type export complex NisFEG (Stein et al. 2003). One line of evidence suggests that NisI intercepts nisin and blocks the peptide from reaching its molecular target lipid II (Stein et al. 2003, Koponen et al. 2004), thereby preventing pore formation (Geiger et al. 2019). Furthermore, recent reports indicate that NisI–nisin interactions also promote cell clustering that acts as a shield to inhibit the action of nisin (AlKhatib et al. 2014). On the other hand, the ABC transporter NisFEG functions by ejecting nisin from the cell membrane before a pore can be formed (Stein et al. 2003). The specificity of NisFEG appears to reside in the C-terminal region of nisin, since deletion of the terminal six amino acids and Ring E reduced the degree of immunity provided by NisFEG (AlKhatib et al. 2014). Although it is postulated that NisI and NisFEG act co-operatively, the role of NisI in self-immunity of the producer appears to be more critical than the transporter since a deletion of *nisI* results in greater susceptibility to nisin compared to a *nisFEG* knockout (Siegers and Entian 1995). While the manner of NisI-mediated immunity is not yet fully understood, recent NMR (Hacker et al. 2015) and molecular modelling studies (Jeong and Ha 2018) have revealed a C-terminal cleft and groove region that may represent important sites for NisI–NisA interactions.

While such immunity or self-resistance systems are typically found in almost all lantibiotic producing strains with individual specificity, it has become apparent that some nonproducers, including pathogenic bacteria, can harbour gene clusters encoding functional proteins and ABC transporters linked to two-component signal TCS (Draper et al. 2015, Clemens et al. 2018, Barbosa et al. 2021). Despite the diversity in their genetic organization, these ‘orphan’ gene clusters confer demonstrable resistance to one or more lantibiotics. Such systems often bear a resemblance to the immunity systems found in lantibiotic gene clusters (LanFEG) or resemble BceAB-type transporters, so-called after the *B*a*c*itracin *e*fflux (Bce) transporter system from *Bacillus subtilis* (Ohki et al. 2003). For example, *Streptococcus mutans* UA159 harbours two systems, one being the LcrSR–LctFEG system that provides resistance to lacticin 481 and nukacin ISK-I and another, NsrFE_1_E_2_G–XRK, that affords resistance to nisin (Kawada-Matsuo et al. 2013). *Clostridioides difficile* (formerly *Clostridium difficile*; Lawson et al. 2016, and from herein termed *C. difficile*) harbours the *cprABCK-R* (*cationic antimicrobial peptide resistance*) operon that provides protection from several lantibiotics including nisin, mutacin 1140 and subtilin (Suárez et al. 2013) and in *L. monocytogenes* the VirSR TCS/VirAB and AnrAB (*abc transporter involved in nisin resistance*) system imparts resistance to multiple antimicrobials including nisin (Kang et al. 2015, Jiang et al. 2019). Moreover, *S. aureus* harbours a complex network of different TCS (BraRS (also known as NsaRS), and GraRS) linked to ABC transporters (VraDE and BraDE) that confer nisin resistance (Blake et al. 2011, Hiron et al. 2011, Randall et al. 2018).

Recently, a resistance operon has been identified in *S. agalactiae* composed of a TCS NsrRK and an ATP-binding cassette transporter NsrFP, but unlike the previously described systems, an additional membrane-associated serine protease termed the nisin resistance protein (NSR) is present (Khosa et al. 2013) that inactivates nisin through proteolytic cleavage at its C-terminus (Sun et al. 2009). The resulting shortened peptide (nisin^1–28^) is up to 100-fold less active and exhibits a considerably reduced ability to form pores (Sun et al. 2009). Furthermore, computational modelling of the protease/nisin complex revealed the importance of the C-terminus of nisin for NSR specificity (Khosa et al. 2013).

The latest studies regarding NsrFP has provided fresh insights into the mechanism of BceAB-type transporters (Gottstein et al. 2022). An unusual feature of these systems is that both transporter permease and the histidine kinase component of the TCS are thought to be mutually indispensable for both sensing of and resistance to the antimicrobial, forming a sensory complex in which the transporter represents the actual sensor (Dintner et al. 2011, Clemens et al. 2018). However, heterologous expression of NsrFP alone (i.e. without its cognate TCS) in *L. lactis* conferred resistance to nisin (Reiners et al. 2017b) and bacitracin, as well as a number of other lipid II targeting compounds (Gottstein et al. 2022). Moreover, comparative proteomic analysis of *L. lactis* cells expressing NsrFP with a nonfunctional mutant (NsrF_H202A_P) suggests that NsrFP may also act by shielding lipid II cycle intermediates from the antimicrobial compound (Gottstein et al. 2022), and thereby provide additional resistance through target protection of cell wall synthesis in agreement with previous studies on BceAB-like systems (Kobras et al. 2020).

Despite its widespread use by the food industry, detection of stable and transmissible resistance to nisin has yet to be reported. However, the presence and distribution of resistance genes as described above across multiple species including important human pathogens poses a significant challenge to potential use of nisin and other bacteriocins in therapeutic applications. For example, the *nsr* operon has been detected in animal and human pathogenic streptococci (including *S. agalactiae, Streptococcus dysgalactiae, S. suis, Streptococcus canis*), staphylococci (including *Staphylococcus capitis, Staphylococcus hyicus, Staphylococcus epidermidis*), and in *Enterococcus faecium* (Khosa et al. 2013, Simões et al. 2016, Field et al. 2018). It is worth noting that these genes are often positioned on transmissible elements such as plasmids (Froseth and McKay 1991, Liu et al. 1997), highlighting the potential for NSR-associated resistance transfer to other microbes. Indeed, the prevalence of NSR amongst lactococci was recently emphasized when whole genome sequencing of 710 dairy-associated *L. lactis* strains found that an impressive 270 (38%) harboured an *nsr* gene (van Gijtenbeek et al. 2021). Furthermore, a singular *nis*I immunity gene located on a 50-kb plasmid was shown to provide nisin resistance to the non-nisin producer *L. lactis* NCDO712 (Tarazanova et al. 2016). These studies highlight the potential for resistance transfer to other genera or species within a shared environmental niche. Ultimately, NGS technologies will facilitate resistance-guided genome mining, which in combination with sequence-based functional metagenomics will not only assist in establishing how prevalent nisin and lantibiotic resistance determinants are within microbiome populations, but also improve the prospects for the discovery of genetic variants or new structurally related homologs of known resistance mechanisms. For example, a whole-genome analysis and evaluation of clinical isolates of *C. difficile* established a link between strains exhibiting high or low nisin resistance to genetic variants in the *cpr*ABC nisin resistance module (Ide et al. 2023). Such knowledge will be invaluable to accelerate the development of strategies that could eventually counteract or avoid nisin resistance action. Several approaches have been investigated in this regard including the application of nisin in combination with other antimicrobials including different bacteriocins and antibiotics, particularly those with different modes of action or that interact synergistically in a bid to target MDR pathogenic targets more effectively (for comprehensive reviews see Mathur et al. 2018, Soltani et al. 2021a). Bioengineering strategies are also being exploited and several engineered variants have been described that can effectively evade some nisin immunity and resistance mechanisms (see later). If nisin is to achieve more widespread therapeutic use, it is critical that resistance be taken into consideration at every stage of development.

## Genome mining and new nisin variants

The exponential increase in genomic data derived from metagenomic sequencing of microbial communities has led to the availability of vast amounts of genetic information to probe for novel lantibiotic operons of interest. The highly conserved sequences inherently found within lantibiotic biosynthetic genes can be utilized as driver sequences to identify areas of a genome that may contain other novel lantibiotic-like BGCs. For example, an *in silico* screen for BGCs with homology to the nisin A biosynthetic genes *nisB* and *nisC* resulted in the identification of more than 49 novel lantibiotic clusters across a range of bacterial species, genera, and phyla not previously linked with lantibiotic production (Marsh et al. 2010). However, a wide variety of genome mining tools have since been developed (BAGEL, AntiSMASH, RiPPMiner, RiPP-PRISM, and RODEO) that have become the preferred and fastest means for the discovery of novel RiPP BGCs (Russell and Truman 2020). Consequently, the availability of high-quality genome sequence data combined with these powerful bioinformatic software packages has greatly expanded our knowledge of the variety and distribution of BGCs, particularly those from human and animal gut microbiomes (Drissi et al. 2015, Walsh et al. 2015). Indeed, given the strengthening association between the gut microbiota and human health and disease (Bull and Plummer 2014), bacteriocin producing strains have attracted significant attention as potential microbiome-shaping tools that could be used in the prevention or treatment of diseases associated with gut pathogens (Mousa et al. 2017, Heilbronner et al. 2021). Genomic mining has doubled the number of natural nisin variants now characterized with the majority of these having been sourced from human, animal, and insect microbiomes. Indeed, only a decade ago, just seven natural variants were known: nisin A, nisin Z, nisin Q, and nisin F produced by *L. lactis* species, nisin U and nisin U2 produced by *Streptococcus uberis*, and a putative nisin P cluster was identified in *Streptococcus pasteurianus* (Field et al. 2015a).

### Nisin H

In 2015, Nisin H was the first variant isolated from a mammalian gastrointestinal tract, in this case, that of a pig (O’Connor et al. 2015). Genome analysis of *Streptococcus hyointestinalis* revealed that it differs from nisin A by five amino acids (Fig. 2B). Despite this, nisin H retains key features of the lactococcal peptides. Another prominent feature was the absence of a corresponding *nisI* immunity gene within the gene cluster (O’Connor et al. 2015).

### Nisin P

Although a nisin P gene cluster was previously noted in the genome of *S. pasteurianus* (Zhang et al. 2012), more recent studies have demonstrated production of nisin P by a clinical isolate of *Streptococcus gallolyticus* (AB39) and the purified peptide displayed antibacterial activity towards several drug-resistant bacteria, including MRSA, VRE, and penicillin-resistant *Streptococcus pneumoniae* (Aldarhami et al. 2020). A nisin P gene cluster was also shown to be present in both a porcine isolate of *Streptococcus suis* (Wu et al. 2014) as well as a strain of *S. agalactiae* isolated from human faeces and was the first such example of a Group B streptococcal strain to produce a nisin variant (Garcia-Gutierrez et al. 2019b).

### Nisin O

*In silico* screening of human gut bacterial genomes identified a nisin BGC in *Blautia obeum* A2–162, a dominant species in the human colon (Hatziioanou et al. 2017). Nisin O is unusual in that it is the first nisin cluster to encode 4 peptides, the first 3 (NsoA1-3) are indistinguishable and resemble nisin U, while the fourth exhibits the greatest deviation from nisin A. Moreover, the gene cluster contains two sets of *nis*RK genes but surprisingly no corresponding *nis*P gene could be identified. The presence of aromatic residues at the first position, as is the case with NsoA1-3 (Fig. 2B), has been shown to significantly reduce cleavage efficiency by the protease NisP (Reiners et al. 2020). The NsoA peptides exhibited strong antimicrobial activity against *C. difficile* and *Clostridium perfringens* following heterologous expression in *L. lactis*, but only in the presence of trypsin (Gherghisan-Filip et al. 2018).

### Nisin BP_SCSK_

**Figure 3.**
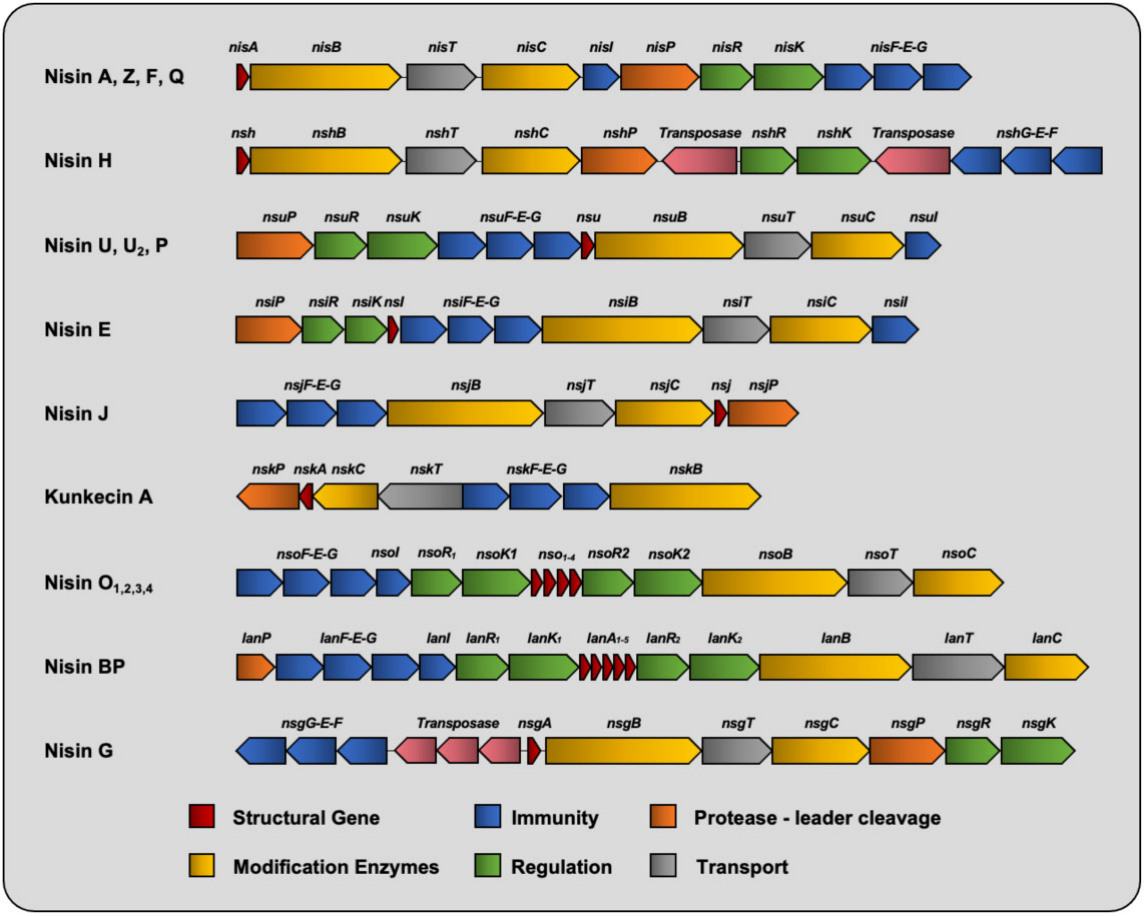
Schematic organization of the BGCs for all nisin variants characterized to date (not to scale).

A multipeptide nisin operon was also present in a *Blautia* species obtained from the faecal microbiota of mice. *Blautia producta* BP_SCSK_ produces a nisin-like peptide variant with similarity to nisin O. In contrast however, BP_SCSK_ encodes five lantibiotic precursor genes (*lan*A1–*lan*A5) (Fig. 3). The first four are identical while the fifth is more divergent. Notably, the antimicrobial activity of BP_SCSK_ was shown to be comparable to nisin A against strains of VRE and other Gram-positive nosocomial pathogens but displayed reduced potency towards other gut commensals (Kim et al. 2019). Nisin BP_SCSK_ differs from nisin A at residues corresponding to I4K, K12V, A15I, G18Dhb, N20P, M21V, K22Q, H27G, and a five-amino acid tail consisting of _29_QIDhbGK_33_ (Fig. 2B). Several of these modifications are located at positions corresponding to bioengineered nisin variants with altered antimicrobial activity and spectrum of antibacterial activity and/or target sites for the digestive enzymes trypsin/α-chymotrypsin including I4, K12, N20P, M21, K22, and S29Q (see later sections on nisin bioengineering/modulation of gut microbiota).

### Nisin J

Nisin J represents the first nisin variant to be produced by a staphylococcal species (O’Sullivan et al. 2020). Identified from the toe web space during a screen of the human skin microbiota, *Staphylococcus capitis* APC 2923 produces a nisin variant with nine residues differing from those in nisin A, as well as having an extra residue at the C-terminus, making it a 35-residue peptide (Fig. 2). Interestingly, some of these amino acid alterations correspond to bioengineered nisin A variants with enhanced activity (see later section on bioengineering), most notably those pertaining to ring A (I4K and L6I) and ring C (M17Q and G18Dhb). Indeed, nisin J was shown to exhibit greater activity against staphylococcal species compared to its A and Z counterparts, prompting the suggestion that the nisin J producer may have naturally evolved to produce a peptide with enhanced activity against other skin-associated staphylococci (O’Sullivan et al. 2020). Unusually, the gene organization of the nisin J cluster also differs from that of other nisin clusters in that the two-component regulatory system (*nisRK*) and the nisin immunity gene *nisI* are absent (Fig. 3).

### Nisin E

The most recent streptococcal-derived nisin variant is nisin E, produced by multiple *Streptococcus equinis* strains, originating from a canine oral cavity as well as sheep gut (Christophers et al. 2023, Sugrue et al. 2023). Nisin E differs from nisin U by two residues (I15A and L21I), but also possesses an extra C-terminal asparagine residue (Asn32). Despite the relatively high homology between the peptides, nisin E displayed reduced activity against a wide panel of target organisms, but most especially against several streptococcal species (*Streptococcus mitis, Streptococus gordonii*, and *Streptococcus anginosus*) compared to its nisin U counterpart, prompting the authors to speculate that the I15A and additional asparagine residue could be responsible (Christophers et al. 2023). Notably, the residues of the hinge region of nisin E (_20_PIK_22_) represent a novel hinge sequence compared to the other closely related nisin U, nisin U2, and nisin P variants (Fig. 2B). Additionally, while the genetic organization of the *nmd* locus essentially matches the nisin U (*nsu*) cluster, the nisin E structural gene (*nmd*A) is situated upstream of the regulatory and immunity modules in contrast to the downstream location of *nsu*A (Sugrue et al. 2023).

### Nisin G

Adding to the growing list of nisin-producing streptococci (*S. uberis, S. hyointestinalis, S. gallolyticus, S. suis, S. agalactiae*, and *S. equinis*) is *Streptococcus salivarius* DPC6487, which was sourced from a neonatal faecal sample and produces nisin G. Although *S. salivarius* strains have been shown to produce another lantibiotic, salivaricin D (Birri et al. 2012), this varies greatly from nisin, distinctly lacking the last MeLan ring. The nisin G peptide diverges from nisin A with respect to seven amino acids (Fig. 2B) and was shown to exhibit a more limited spectrum of activity when compared to nisin A, with activity confined to other streptococci but more notably to *Fusobacterium spp*, including *Fusobacterium nucleatum. Fusobacterium nucleatum* is an emerging human pathogen linked to several gut-associated disorders including colorectal cancer (CRC) development. The desire for intestinal-derived bacteriocin producing strains that can kill specific target organisms without causing collateral damage to host bacterial populations makes *S. salivarius* DPC 6487 a potential candidate for probiotic development (Lawrence et al. 2022).

### Kunkecin A

The nisin variant Kunkecin A (Zendo et al. 2020) was identified from a honeybee isolate *Apilactobacillus kunkeei* FF30-6, a fructophilic lactic acid bacterium recently characterized as one of the major components in the gastrointestinal tract of honeybee queens and larvae (Endo and Salminen 2013). Kunkecin A represents the longest natural variant described to date, possessing five extra amino acids at the C-terminus compared to nisin A (Fig. 2B). Despite displaying an overall narrow antimicrobial spectrum, kunkecin A was reported to exhibit superior activity over nisin A against several bacteria originating from honeybees, including *Melissococcus plutonius*, the causative agent of European foulbrood, a global honeybee brood disease. Crucially, many other honeybee commensals including lactobacilli and bifidobacteria appeared less sensitive to this nisin variant, prompting the authors to suggest the kunkecin A producer as a potentially useful probiotic to inhibit honeybee pathogens in apiaries.

There is mounting evidence that bacteriocins produced by microbial residents of the gut impart a competitive advantage and play a major role in shaping niche competition among intestinal bacteria (Dobson et al. 2012). While some bacteriocins display a narrow range of activity, targeting only closely related members of the same species, others like nisin display a broader spectrum of activity. Recent microbiome investigations have begun to elucidate the impact of nisin on the gastrointestinal microbiota (outlined below) and reveal in more detail members of the microbiome that are susceptible or resistant to its action. Given the contrasting antimicrobial activities of the natural nisin variants when compared to nisin A as discussed above, it is tempting to speculate that these variants have evolved because of localized competition with specific microbes in their respective environments. Indeed, the potential for more precise targeting of individual pathogens whilst at the same preserving the integrity of the microbial composition are very desirable properties in light of the important role of the microbiome in overall human health.

## Nisin bioengineering to modulate antimicrobial activity and spectrum of antibacterial activity

The inexorable proliferation of MDR pathogens has significantly impacted on the effectiveness of commonly used antibiotics (Gupta and Datta 2019). Consequently, there is an urgent need for new antimicrobial compounds as well as novel derivatives of current antimicrobials that specifically target MDR bacteria and/or other problematic organisms. A consequence of the gene encoded nature of nisin is the relative ease with which new structural variants can be created through genetic manipulation. Moreover, the diversity of the natural variants and related homologues emphasizes the tolerance to changes of particular residues and regions within the peptide. Indeed, their activity and physicochemical properties would suggest they have evolved to kill specific or a narrower range of targets, and thus could be viewed as templates for new, tailor-made and specific targeting peptides.

Over the last two decades, banks of engineered nisin derivatives have been generated and characterized. Simple single or multiple modifications and chimeric molecules are beginning to furnish a blueprint of those residues and domains essential for structure–activity relationships—not only relating to nisin biosynthesis, but also in terms of antimicrobial activity and spectrum, solubility, heat stability, challenges to immunity or resistance proteins as well as sensitivity to proteolytic enzymes. The following section will focus on recent advances regarding the implementation of bioengineering strategies to enhance the functional characteristics of nisin and the most notable successes achieved as a consequence of employing these various strategies. For prior bioengineering studies, we direct the reader to a number of comprehensive reviews (Lubelski et al. 2008, Field et al. 2015a, Shin et al. 2016).

Nisin can be structurally divided into an N-terminal region (composed of a lanthionine ring A and the methyllanthionine rings B and C, a hinge region, and a C-terminal region; with the intertwined methyllanthionine rings D and E) (Fig. 2A). The impact of mutagenesis on each of these regions will be discussed in terms of improved functional characteristics.

### N-terminus

**Figure 4.**
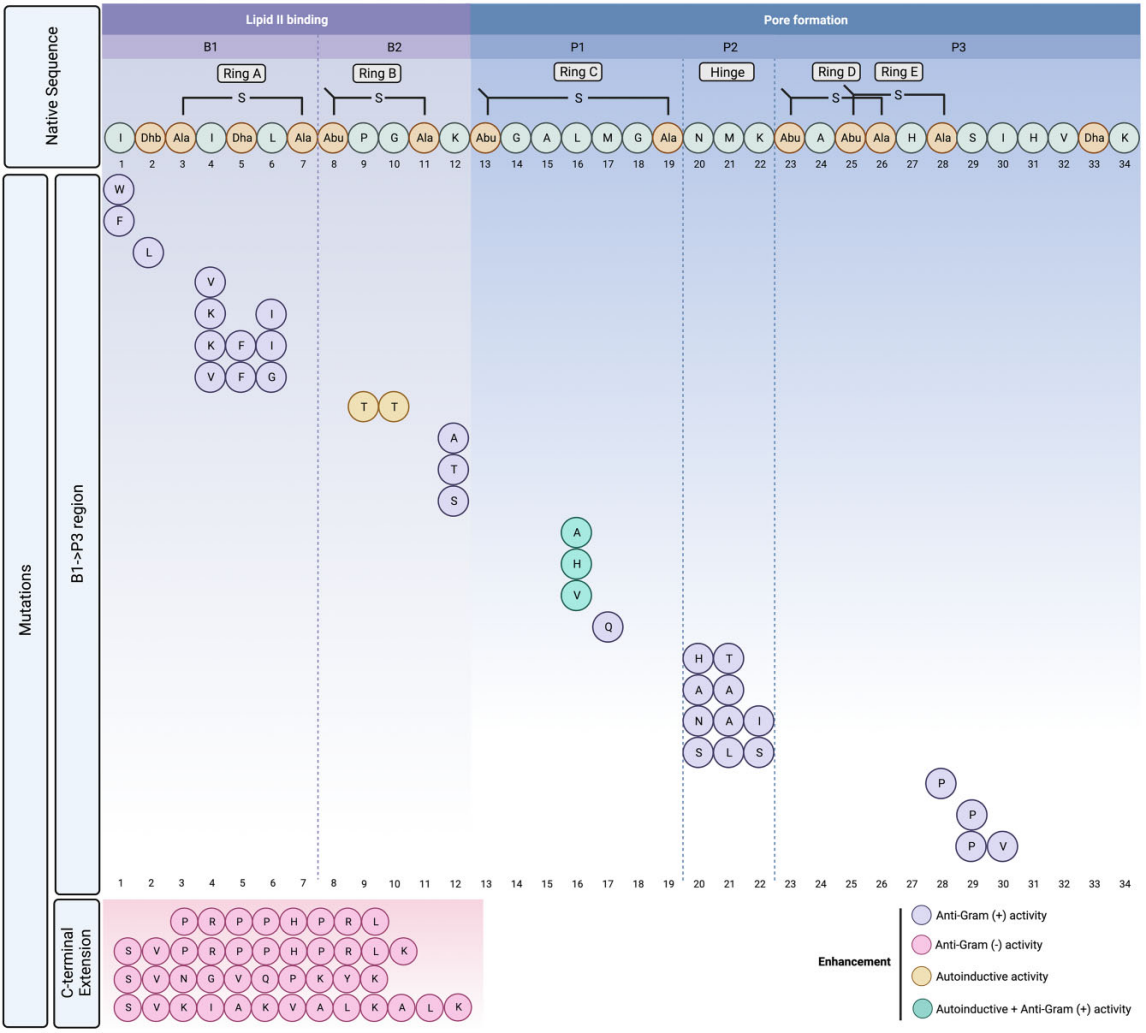
Summary of enhanced bioengineered nisin derivatives. Variants with increased specific activity are denoted by purple and pink for Gram-positive and Gram-negative bacteria, respectively. Enhancement of autoinduction properties are denoted by yellow circle or enhancement of both activity and autoinduction by green circle. A blueprint of nisin with the Lan/MeLan Rings A–E and the hinge region is interpreted as five distinct modules B1–P3 according to the sequence B1/B2/P1/P2/P3 representing a lipid II binding domain, a flexible domain, and a pore-forming domain (Schmitt et al. 2019). Post-translational modifications in the native nisin A sequence are indicated as follows: Abu: 2-aminobutyric acid, Ala-S-Ala: lanthionine, Abu-S-Ala: 3-methyllanthionine) Dha: dehydroalanine, and Dhb: dehydrobutyrine.

A systematic saturation mutagenesis approach at the N-terminal isoleucine (Ile1) and analysis of the resulting 20-generated analogues revealed that although the core peptide was completely modified, impacts on production, leader peptide cleavage, and antimicrobial activity varied drastically and correlated with the nature of the amino acid substituted in each case (i.e. aliphatic, aromatic, charged, and so on) (Lagedroste et al. 2019). Results revealed that aromatic amino acids at position one (I1W and I1F) gave rise to superior antimicrobial activity (Fig. 4), particularly against lactococcal strains expressing the nisin immunity and resistance proteins nisI, nisFEG, NSR, and NsrFP. In contrast, polar or charged amino acids triggered a reduction in activity, highlighting the influence small changes in the peptide structure can have on activity and ability to circumvent nisin resistance systems. Moreover, the majority of Ile1 variants were subject to NisP processing albeit with lower efficiency, the exception being I1P, which remained intractable to NisP activity.

As one of the earliest locations targeted for mutagenesis (Kuipers et al. 1995, Wiedemann et al. 2001), position two variants provided the first evidence that nisin activity could be improved when the threonine at position 2 in nisin Z (a residue, i.e. normally converted to dehydrobutyrine) was changed to a serine/dehydroalanine and displayed enhanced activity against two nonpathogenic target organisms (Kuipers et al. 1995). More recent investigations have highlighted the importance of Thr2 within the lipid II binding motif of nisin when a bioengineered chimeric lantibiotic found to be more potent against VRE was rendered inactive following a T2D substitution (Zhao et al. 2020b). Notably, in a targeted approach to identify nisin peptides more suited for bovine mastitis applications, a threonine to leucine variant (T2L) (Fig. 4) was shown to exhibit exceptional antimicrobial activity against a selection of bovine mastitis-associated staphylococci (Field et al. 2021), but were noticeably less active against many of the commensal lactic acid bacteria found in milk such as lactococci and lactobacilli.

### Rings A and B

Early engineering attempts targeted at the N-terminal ring A concerned a S3T substitution (which would change the first Lan residue to MeLan) that led to a dramatic loss of activity (Wiedemann et al. 2001). The amino acid composition of ring A appears quite variable as is evident upon inspection of the contrasting ring arrangements in several natural nisin variants (Fig. 2B) and nisin-like peptides. This diversity at positions 4–6 is in a region, i.e. at the border of the pyrophosphate cage involved in the mechanism of action of nisin (Hsu et al. 2004), underscoring the large mutational freedom available and may thus represent suitable targets for mutagenesis. Indeed, two variants generated via saturation mutagenesis at positions 4–6 corresponding to _4_KSI_6_ and _4_KFI_6_ (Fig. 4) displayed improved activity against several nonpathogenic indicator strains (Rink et al. 2007). Notably, several natural nisin variants as well as novel nisin-like peptides (agalacticin, flavucin, moraviensicin, and maddinglicin) (van Heel et al. 2016) possess a lysine at position 4 (I4K) (Fig. 2B). Additionally, a _4_VFG_6_ derivative retained strong antimicrobial activity but suffered a dramatic loss in its autoinduction capacity. Moreover, these variants had the ability to escape the self-immunity proteins of NisI and/or NisFEG, proving to be toxic to a nisin producing strain (Rink et al. 2007). More conserved residue changes such as the nisin I4V variant (Fig. 4) demonstrated improved antimicrobial as well as potent antibiofilm activity against several strains of *Staphylococcus pseudintermedius* (Field et al. 2015b). These enhanced antimicrobial properties were further extended to include *S. aureus* when I4V was combined with conventional antibiotics (Field et al. 2016).

The highly conserved ring B is composed of a MeLan and the amino acids proline (Pro9) and glycine (Gly10). Mutational analysis has underpinned its importance in both antimicrobial and induction activity (Rink et al. 2007, Ge et al. 2016). For example, the _9_PT_10_ variant retained comparable induction capacity and antimicrobial activity as nisin A, while _9_PH_10, 9_PR_10, 9_PD_10, 9_PN_10_, and _9_PL_10_, displayed significantly lower induction and antimicrobial activities due to incomplete ring formation (Rink et al. 2007). More recently, a ring B variant where both residues are replaced with threonine (_9_TT_10_) (Fig. 4) and designated nisin M, retained full autoinduction capability but exhibited up to more than 16-fold less activity against several genera and species of bacteria (O’ Connor et al. 2020a), emphasizing the lack of a direct correlation between lipid II binding and induction capacity.

The residue Lys12 is located between rings B and C and a site-saturation approach at this location led to the identification of a small number of bioengineered derivatives with improved activity (Molloy et al. 2013) (Fig. 4). Indeed, more recent NMR analyses confirmed the importance of Lys12 as a ‘pharmaceutical hotspot’ by acting as a flexible region that permits nisin to adopt a specific conformation within the bacterial membrane environment (Medeiros-Silva et al. 2018).

### Ring C

Although the precise function of ring C has not yet fully been elucidated, this MeLan ring has been shown to be critical for the biological activity of nisin (van Kraaij et al. 2000) and suggests that this region is involved in very specific interactions. For example, converting the thioether bond of ring C to a disulphide bond was found to abolish activity completely (van Kraaij et al. 2000). Recent studies have revealed the unique membrane-interacting properties of ring C and have implied a structural link between the arrangement of the hinge in tandem with ring C conformations in the ability to form pores (Medeiros-Silva et al. 2018). Mutational analysis of residues within ring C has been beneficial with respect to enhanced functional derivatives as was observed by L16A, L16H, and L16V (Fig. 4) that displayed a slight increase in both antimicrobial activity and induction capacity (Ge et al. 2016). More recent reports have demonstrated the enhanced specific activity of other ring C variants against pathogenic strains, as when nisin M17Q proved to be more effective than nisin A at reducing *Staphylococcus epidermidis* biofilms from medical device-related materials as well as significantly reducing viable cells in simulated wound fluid experiments (Twomey et al. 2020). Nisin M17Q (Fig. 4) was also shown to exhibit enhanced activity against some strains of bovine mastitis-associated *S. aureus* (Field et al. 2021) and displayed antilisterial activity when used in combination with other bioengineered nisin derivatives (Nyhan et al. 2021), which was sustained in model food experiments.

### The hinge region

The hinge consists of a 3-amino acid linker region, i.e. critical for antimicrobial activity by providing conformational flexibility between the N- and C-termini of nisin. Following interaction between the two N-terminal rings with lipid II, the flexible hinge region facilitates insertion of the nisin C-terminal domain into the bacterial membrane to form a pore (Breukink et al. 1999, Wiedemann et al. 2001).

Bioengineering of the hinge region provided the first reports of derivatives with improved activity against Gram-negative pathogenic targets (Yuan et al. 2004), which was followed shortly thereafter by improved variants against Gram-positive organisms (Field et al. 2008). An approach involving simultaneous randomization of all three hinge residues in nisin A (_20_NMK_22_) generated a suite of peptides whereby a preference for small, chiral amino acids was linked to increased bioactivity (Healy et al. 2013). Additionally,s a nisin peptide incorporating a hinge region composed of _20_HTK_22_ (Fig. 4) represents a novel variant (Field et al. 2021) to add to those previously identified as having improved antimicrobial activity (_20_AAK_22, 20_NAI_22_, and _20_SLS_22_) (Healy et al. 2013).

Furthermore, the impact of expanding or reducing hinge length on the antimicrobial activity and target spectrum of nisin has also been explored. Zhou et al. (2015) revealed that both shortened hinge peptides (−1 amino acid) and extended hinge peptides (+2 amino acids) displayed improved efficacy as determined by growth inhibition assays against several target strains including *L. lactis, Enterococcus faecalis, L. monocytogenes, and B. cereus*, but were target organism- and temperature-dependent due to variations in bacterial membrane composition. Similarly, Zaschke-Kriesche et al. (2019b) examined the advantage of decreasing (Δ_21_MK_22_) or extending the hinge region (_20_NMKIV_24_ and _20_NIVMK_24_). These hinge variants were impacted in their ability to form pores when compared to wild type nisin A, in particular the truncated Δ_21_MK_22_ peptide, and when assessed against strains expressing the nisin immunity (NisI and NisFEG) and nisin resistance determinants (SaNSR and SaNsrFP), the variant _20_NMKIV_24_ displayed increased activity, possibly as a result of reduced substrate recognition (Zaschke-Kriesche et al. 2019b)

### C-terminal rings and tail

The C-terminus of nisin A is essential for pore formation and consists of the highly conserved rings D and E followed by a six-amino acid tail (_29_SIHVDhbK_34_). NMR studies have revealed Ser29 within this region as an important flexible region for the orientation of nisin within the membrane pore (Medeiros-Silva et al. 2018). Indeed, a site-saturation approach revealed that bioengineered mutants at this location extended nisin activity towards some Gram-negative species (Field et al. 2012).

Recent investigations have highlighted the importance of ring D and E for recognition by the NSR (Khosa et al. 2016a). Specifically, the resistance provided by NSR was overcome by nisin variants lacking rings D and E or only ring E. This was further emphasized by molecular simulations of the NSR/nisin complex, which revealed the role of the C-terminal rings for substrate specificity to ensure the exact coordination of the nisin cleavage point (Ser29) at the enzymatic active site (Khosa et al. 2016b). Considering this, Field et al. (2018) used saturation mutagenesis such that serine at position 29 was replaced with all other 19 amino acids. The results identified one derivative, S29P (Fig. 4), as having comparable activity to nisin A but crucially exhibited a 20-fold increase in specific activity against several NSR producing strains due to the inability of NSR to cleave the mutant peptide. Furthermore, another variant termed nisin PV (S29P and I30V substitutions)) proved to not only be as active as S29P, but to be more stable by virtue of being less prone to oxidation (Field et al. 2018). Alternatively, replacement of Cys 28 with proline (C28P) resulted in a nisin A peptide that was lacking ring E and retained a more structurally rigid and smaller ring (Zaschke-Kriesche et al. 2019a). Nisin C28P (Fig. 4) was as active as wild type nisin and retained the ability to form pores in the membrane, but was notably more effective against an NSR producing strain due to the inability of NSR to cleave this variant efficiently (Zaschke-Kriesche et al. 2019a).

There are just a handful of novel antibiotic compounds in current development that target Gram-negative bacteria (Imai et al. 2019). The outer membrane of Gram-negative organisms is impermeable to nisin, preventing access to the inner membrane and its target lipid II (Nikaido and Vaara 1985). Consequently, nisin exhibits poor activity towards Gram-negative species. However, disruption of the outer membrane with chelating agents such as EDTA can restore susceptibility to nisin (Stevens et al. 1991). Thus, the main impediment for nisin to kill Gram-negative bacteria appears to be its inability to traverse the outer membrane. A recent approach to assist nisin in penetrating the outer membrane involved the fusion of several small anti-Gram-negative peptides tails including apidaecin 1b, oncocin, and drosocin to the C-terminal end of nisin (Zhou et al. 2016). One such fusion, containing an eight amino acid (PRPPHPRL) tail from apidaecin 1b attached to full length nisin (Fig. 4), exhibited 2-fold greater activity against *Escherichia coli* CECT101, highlighting that the strategy of combining a lipid II binding peptide with the penetrative ability of eukaryotic antimicrobial peptides extends the activity of nisin towards Gram-negative bacteria (Zhou et al. 2016). Subsequent studies involved a greater selection of peptide tails and re-engineered variants and identified several of them with greater activity against a range of important pathogenic Gram-negative organisms including *E. coli, Klebsiella pneumoniae, Acinetobacter baumannii, Pseudomonas aeruginosa*, and *Enterobacter aerogenes* when compared to nisin alone (Li et al. 2018). Moreover, bioengineering approaches to include different linker domains as well as a C-terminal supplementary lysine residue (to mimic many lantibiotics that possess a positively charged amino acid at the C-terminus) proved beneficial in more effectively targeting Gram-negative pathogens (Fig. 4).

### Bioengineering modular nisin analogues and semisynthetic hybrids

**Figure 5.**
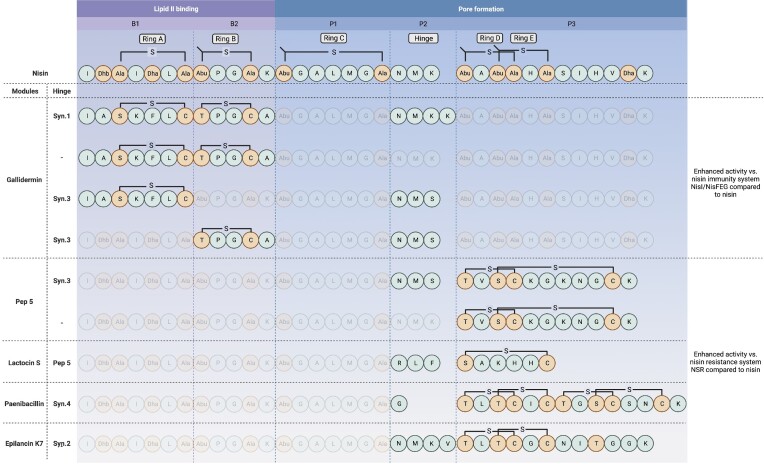
Enhanced variant peptides derived from the molecular shuffling of functional modules from nisin and 12 different class I and class II lantibiotic peptides (adapted from Schmitt et al. 2019). A blueprint of nisin with the Lan/MeLan Rings A–E and the hinge region is interpreted as five distinct modules B1–P3 according to the sequence B1/B2/P1/P2/P3 representing a lipid II binding domain, a flexible domain, and a pore-forming domain).

More than 100 lanthipeptides have now been identified that exhibit enormous structural diversity; especially in terms of post-translational modifications including thioether-based intramolecular rings, unusual dehydroamino and unsaturated amino acids as well as a variety of flexible linker regions that when combined, are critical for stability and biological activity (Dischinger et al. 2014). Despite this diversity, a common feature of lantibiotics is the ability to bind lipid II and form pores in the membrane, a blueprint that encompasses different functional elements or modules. Importantly, lantibiotic peptides vary substantially in terms of activity towards target strains. While bioengineering strategies have been crucial for advancing our knowledge with respect to structure–activity relationships and have produced derivatives with enhanced properties, in the main these studies have focused on the diversification of individual lantibiotic peptides which can deliver only limited structural novelty. A recent study sought to investigate the consequences of the molecular shuffling of functional modules (i.e. a lipid II binding domain, a flexible domain, and a pore-forming domain) from 12 different and well-characterized class I and class II lantibiotic peptides including nisin, gallidermin, Pep5, Lacticin 481, mersacidin, and cinnamycin that also included synthetic linker modules to imitate or replace the hinge region of nisin (Schmitt et al. 2019). A library of over 6000 combinatorial variants was generated and attached to the leader peptide of nisin and expressed using the nisin biosynthetic machinery NisBTC in *L. lactis*. Following library screening, 11 new to nature peptides were found to exhibit improved antimicrobial activity compared to their wild-type counter parts against a panel of Gram-positive pathogens including *S. pneumoniae*, MRSA, VRE (*E. faecalis* and *E. faecium*) as well as *L. lactis* strains harbouring the nisin immunity cluster (*NisIFEG*) and the NSR. Notably, peptides consisting of combinatorial modules of gallidermin and nisin joined by a synthetic hinge were most active against the majority of pathogenic strains tested as well as being able to bypass the nisin immunity system (Fig. 5). Additionally, peptide derivatives containing atypical hinge and C-terminus modules (such as those found in Pep5, lactocin S, paenibacillin, and so on) proved to be more active against NSR cleavage, highlighting the importance of the C-terminus of nisin for NSR recognition (Schmitt et al. 2019) (Fig. 5).

The targeting of lipid II, crucial for bacterial cell wall synthesis and readily accessible on the outer surface of the cell membrane, is an effective and ancient antimicrobial strategy (Ulm and Schneider 2016) and an important target for potent antibiotics including vancomycin, teixobactin, and several lantibiotics. Rings A and B contain the lipid II binding motif of nisin, i.e. conserved among other lantibiotics including subtilin, epidermin, gallidermin, microbisporicin, and mutacin 1140, and have been the focus of several strategies to generate novel lipid II targeting compounds (Koopmans et al. 2015, Deng et al. 2020, Zhao et al. 2020b).

One such approach involved the chemical coupling of functional moieties to N-terminal nisin ring fragments (AB, ABC ring systems) using click chemistry. The addition of membrane active lipids to the nisin AB ring fragment yielded semisynthetic analogues with enhanced stability (Koopmans et al. 2015) while the conjugation of synthetic hydrophobic polyproline peptides to rings ABC produced nisin hybrids that displayed increased antimicrobial activity against *E. faecium* when compared to the nisin ABC fragment alone, though they proved to be 8-fold less active than full length nisin (Deng et al. 2020). Moreover, two nisin hybrids could bypass the NSR and were less prone to proteolytic degradation.

In another approach, novel hybrid peptides were designed consisting of two different lipid II binding regions, one composed of the N-terminal region fragments of nisin (1–10, 1–11, 1–17, 1–25, and so on) linked to the C-terminal lipid II binding regions of the two-component lantibiotics lacticin 3147 or haloduracin (Zhao et al. 2020b). A suitable linker region was included between each of the domains. Of 20 such hybrid peptides that were expressed using the nisin biosynthetic machinery, two showed potent antimicrobial activity against *Micrococcus flavus*. Further analysis of one such hybrid, TL19 (composed of nisin + haloduracin lipid binding motifs), displayed 64-fold higher potency against *E. faecium* compared to the single lipid II binding component of nisin1-22, highlighting the potential of a single molecule with two lipid II binding motifs to enhance antimicrobial activity (Zhao et al. 2020b).

Recently, a cell-free protein synthesis (CFPS) system using *E. coli* cell extracts was employed for the production of novel variants of nisin Z (Liu et al. 2020). This required some adjustments such as increasing the levels and ratio of NisB and NisC in conjunction with the precursor peptide for optimal efficiency. Following a search of all publicly available genomes for nisin analogues, 18 novel core peptide variants were subsequently linked to the nisin leader peptide and expressed in the CFPS system. In total, four variants displayed antimicrobial activity and following further purification exhibited good activity against *E. faecalis, S. aureus*, and MRSA, with the RL14 variant exhibiting superior activity compared to nisin Z against *E. faecalis*. Notably, the residue changes across the four peptides included I4K and I4V, K12V, A15V and A15I, M17Q, and G18Dhb, a shortened hinge (_20_AL_21, 20_AI_21_, and _20_PI_21_) and a varied C-terminal tail. An additional library of 3000 variants was generated via saturation mutagenesis targeting positions 4, 12, 15, 24, and 29 of nisin Z and expressed in the CFPS system. Subsequent screening identified a further two variants with improved activity against Gram-negative bacteria compared to parental nisin Z (Liu et al. 2020).

In a bid to identify novel peptides that specifically target *C. difficile*, a *Clostridium* genome mining approach was employed that screened over 600 publicly available genomes. A total of 10 putative lanthipeptide genes were identified in *Clostridium beijerinckii, Clostridium ihumii*, and *C. perfringens* and expressed in *L. lactis* using the nisBCT synthetase (Cebrián et al. 2019). When all but one failed to produce antimicrobial activity, a synthetic biology approach was undertaken whereby a new series of hybrid lantibiotics were designed and expressed that were composed of N-terminus or C-terminus modules of the putative clostridial peptides fused to the N-terminus or C-terminus modules of nisin. An active hybrid peptide with good antimicrobial activity against a *C. difficile* strain was isolated but was notably less active against other clostridial strains. Interestingly, the peptide was not active against *C. beijerinckii*, the strain from which the N-terminus module used in the hybrid was composed (Clos AB + nisin CDE). The authors note that the specificity and activity observed for the hybrid peptide makes it an interesting potential candidate in the treatment of *C. diffic*ile infections, avoiding side effects and protecting the normal gut microbiota (Cebrián et al. 2019).

## Nisin as a modulator of the gut microbiota

It is now becoming increasingly clear that the gut microbiome is integral to human health (Mousa et al. 2017, Garcia-Gutierrez et al. 2019a). Indeed, maintaining a healthy gut microbiome could be considered as a new therapeutic target since any significant imbalance could contribute to the development or progression of diseases such as inflammatory bowel disease, diabetes, obesity and infection by intestinal pathogens such as *C. difficile* (Duan et al. 2022). Enteric diseases in farm animals are also a cause of significant economic losses in the agribusiness sector. Furthermore, treating such diseases accounts for considerable antibiotic use in both humans and animals that not only has the potential to adversely affect the composition and function of the gut microbiome, but also encourages the selection of antibiotic-resistant pathogens. It would, therefore, be advantageous to be able to modulate the gut microbiota to selectively target or deplete undesirable microbes without impacting the beneficial microbes. Bacteriocins are gaining credibility as precise modulators of the human microbiome (O’Connor et al. 2020b). Indeed, several recent studies provide comprehensive evidence that bacteriocins and bacteriocin-producing bacteria can be used to modify the gut microbiota, making them an attractive strategy to address gut-related diseases and disorders (Guinane et al. 2016, Umu et al. 2016, 2017, Lawrence et al. 2022). Bacteriocins could address some of the issues associated with conventional antibiotics and significant research is ongoing to elucidate any role they could play within the gastrointestinal tract, their ability to inhibit pathogens as well as any potential functions in safeguarding host health (Heilbronner et al. 2021). Several recent studies have investigated the capacity of nisin, either by direct application or secreted by *in situ* bacteria, to influence the gut microbiota and/or to control specific pathogens associated with chronic intestinal diseases. For example, it was evident that nisin, when supplemented in the diet of poultry, can beneficially modulate the gastrointestinal ecology and enhance growth performance (Józefiak et al. 2013, Kieronczyk et al. 2017, 2020). Similar effects were observed in rabbit models (Lauková et al. 2014). Likewise, nisin treatments elicited significant changes in the gastrointestinal microbiota in a bacterial diarrhoea mouse model by increasing favourable species such as *Lactobacillus, Bacteroides*, and *Bifidobacterium* while reducing pathogenic strains of *E. coli* and *Enterococcus* spp (Jia et al. 2018). More recently, when nisin was used as a feed additive in aquaculture it was shown to alter the diversity and composition of the intestinal microbiota of common carp (Ke et al. 2021), bream (Moroni et al. 2021), and flounder (Nguyen et al. 2018).

Nisin has also been considered for its therapeutic potential in targeting the gut pathogen *C. difficile*. Antibiotic use is a major risk factor for developing a *C. difficile* infection as a result of changes to the gut microbiota that allow *C. difficile* to proliferate and cause recurrent *C. difficile*-associated diarrhoea and intestinal inflammatory disease (collectively designated *C. difficile* infection or CDI). The standard treatment for CDI is use of the antibiotics fidaxomicin, metronidazole, or vancomycin, although none of these antibiotics are fully effective (Czepiel et al. 2019). In comparative studies carried out with nisin, vancomycin, and metronidazole against >60 clinical *C. difficile* isolates, the results revealed that nisin was the most effective of the three antimicrobials tested. Nisin was more potent as observed by its overall MIC90 of 0.256 mg/l while both vancomycin and metronidazole had MIC90s of 0.8 mg/l (Bartoloni et al. 2004). Furthermore, nisin A was shown to significantly reduce *C. difficile* spore viability in liquid suspension following 1 hour of treatment (Lay et al. 2016).

To evaluate nisin in conditions that more closely mimic the GIT and as a consequence observe the effects on the total microbiota, researchers have explored its ability to inhibit or kill *C. difficile* in model colon systems (Le Lay et al. 2015, O’Reilly et al. 2022). Nisin A (in the form of Nisaplin^®^) proved to be an effective inhibitor of *C. difficile*, achieving over a 100-fold reduction in cell numbers when used at 76 µmol/l (equivalent to 20X MIC) in a simulated human colon system (consisting of a cell immobilized and continuous fermentation single-stage reactor to simulate the proximal, transverse, and distal colons; Cinquin et al. 2004, Le Lay et al. 2015). The treatment also brought about significant alterations to the microbial composition whereby Gram-positive bacteria were notably disturbed. Indeed, *Ruminococcaceae* were most impacted in that they underwent an almost 4-log reduction, while *Lachnospiraceae, Lactobacillaceae, Leuconostocaceae* groups as well as bifidobacteria were less affected (Le Lay et al. 2015). Although an increase in the Gram-negative population (*Bacteroidetes* and *Enterobacteriaceae)* was also noted, the initial balance was restored 24 hours after nisin addition. In the same study, a *L. lactis* nisin producer, which was shown to persist and proliferate in the model colon, was ineffective against *C. difficile*, most likely due to the inability of the strain to generate a sufficient quantity of peptide in these conditions to inhibit the pathogen (Le Lay et al. 2015).

A recent report employed the micro-Matrix 24-well parallel controlled cassette minifermentation system as a batch colon model (as described by O’Donnell et al. 2018) to investigate the efficacy of pure nisin A to kill *C. difficile* in a dose-dependent manner. Importantly, it was established that a concentration of 50 µM was sufficient to completely eradicate *C. difficile* whilst simultaneously eliciting the least impact on the overall microbial composition (O’Reilly et al. 2022).

*Enterococcus* spp are also commonly found in the human GIT (Vankerckhoven et al. 2004). Worryingly, some enterococci, in particular *E. faecalis* and *E. faecium* are a leading cause of hospital-acquired infections, as a result of developing resistance to many frontline antibiotics including vancomycin (Miller et al. 2014). Antibiotic administration disrupts the normal gut microbiota balance, leading to expansion of VRE in the intestinal tract, predisposing vulnerable patients to bloodstream infections. The situation is so challenging that VRE have been earmarked by the World Health Organization as a critical target for the identification and development of new antimicrobials and novel approaches to combat infections (AbdelKhalek et al. 2018). Notably, a recent study revealed that administration of a four strain cocktail consisting of *Clostridium bolteae, Bacteroides sartorii, B. producta*, and *Parabacteroides distasonis* could reduce the density of enterococci in the colon of infected mice and restore resistance to VRE infection (Caballero et al. 2017). One of the consortia, *B. producta* (BP_SCSK_) was found to inhibit VRE by production of the nisin variant, nisin BP_SCSK_. Moreover, this variant exhibited reduced activity against intestinal commensal bacteria including *Bifidobacterium longum* and *Pediococcus acidilactici* (Kim et al. 2019). Notably, substitution of the nisin BP_SCSK_ producer for a *L. lactis* nisin producing strain in the consortium failed to inhibit VRE *in vivo*, most likely as a result of its inability to colonize the gut. This study elegantly demonstrates that nisin expression by a commensal microorganism can influence niche competition in the gastrointestinal tract by preventing pathogen colonization and with minimum disruption to the microbiota.

A major limitation with respect to the therapeutic use of nisin is its sensitivity to proteolysis by intestinal enzymes. Nisin A has been shown to be degraded by pepsin, trypsin, and chymotrypsin (Heinemann and Williams 1966, Jarvis and Mahoney 1969, Slootweg et al. 2013). More recent investigations involving simulated oral, gastric, and small intestinal digestion trials also revealed the proteolytic degradation of nisin A (Gough et al. 2017) and nisin Z (Soltani et al. 2021b). Indeed, despite the low pH environment (pH 3), nisin Z remained mostly intact under gastric conditions with only minor degradation fragments detected, but the peptide was significantly degraded after 2 hours under conditions representing the small intestine (pH 7). Moreover, in addition to some of the expected breakdown products of proteolysis, oxidized forms of nisin were detected in the untreated, oral and gastric fractions (Soltani et al. 2021b), most likely from oxidation of the methionine residue (M21) located in the hinge region of the peptide (Rollema et al. 1996). Previous findings have shown that the degree of oxidization in nisin peptides leads to a reduction in antimicrobial activity (Yoneyama et al. 2008), highlighting other potential factors that could play a role in the efficacy of nisin.

In order to bypass the sensitivity of nisin to proteolytic cleavage, encapsulation has been investigated as a means of facilitating transit through the GIT. Indeed, *in vitro* studies have found that a pectin/HPMC (hydroxypropyl methyl cellulose) envelope is suitable for delivery of nisin to the colon (Ugurlu et al. 2007). Similarly, encapsulation of nisin using two different resistant starch-based matrices was investigated for delivery to the lower GIT and its release in a controlled manner. Analysis of faecal pellets of mice fed the encapsulated nisin revealed the presence of fully intact and functional peptides, though the concentration detected was matrix dependent (Gough et al. 2018). Finally, a variety of silica-based mesoporous matrices were examined for nisin encapsulation and found to protect nisin A from the proteolytic action of pepsin in biorelevant media, highlighting the suitability of this approach to shield nisin from any degrading enzymes in the intestinal tract (Flynn et al. 2019). Other novel means of providing proteolytic resistance could take the form of the chemical coupling of specific synthetic and nonproteinaceous moieties to nisin that can provide the desired resistance to specific proteases (Deng et al. 2020).

Interestingly, nisin A contains three trypsin sensitive residues; K12, N20, and K22 while N20, M21, and H31 are chymotrypsin sensitive residues (Slootweg et al. 2013). Thus, it is notable that many of the amino acids corresponding to trypsin/α-chymotrypsin ‘target’ sites i.e. K12, N20, M21, and H31 have been replaced in the gut-derived natural nisin variant nisin O, and are completely replaced with nontarget residues in nisin BP_SCSK_, (K12V, N20P, M21V, K22Q, and H31Dhb), a response that suggests adaption to their environment in the gut. Moreover, the reduced activity of nisin BP_SCSK_ against some beneficial commensal bacteria suggests these residues are excellent targets for bioengineering not only to maintain protection from disintegration in the gastrointestinal tract but also to enhance activity towards specific pathogenic organisms whilst having minimum impact on the commensal microbiota resident in the gastrointestinal tract.

## Perspectives

In the face of the antimicrobial resistance crisis, researchers are struggling to identify new antibiotic classes. Nisin has been studied since the earliest days of the antibiotic era, and although it has found global success as a biopreservative, its multiple modes of action, potent activity against MDR microbes and long safety record has meant that the focus on nisin-related research is shifting from food preservation towards therapeutic use for the treatment of bacterial infections.

The modular nature of nisin and other lantibiotics and the development of expression systems to reorder the extensive range of thioether-derived ring structures at random (akin to a plug ‘n’ play system with provision for an almost inexhaustible array of structural permutations) is a thrilling new prospect for nisin and lantibiotic research. This, and advances in widening the substrate specificity of existing modification enzymes will undoubtedly lead to new structures with enhanced functional characteristics (specific activity, target spectrum including Gram-negative targets, diffusion, solubility, and improved gastrointestinal stability) and with them a portfolio of potential therapeutic applications.

The discovery and characterization of new natural variants from different environments, including the gastrointestinal tract of humans and animals, is welcome and likely to accelerate with the advent of cheaper and faster genome sequencing technologies and finely tuned genome mining tools. Indeed, the current rate of discovery emphasizes the broad distribution of nisin-related BGCs across bacterial species, implying a strong role in Gram-positive bacterial competition within a variety of microbiomes. Furthermore, the mounting evidence of the beneficial role of the gut microbiome in human and animal health will ensure that greater efforts will focus on the ability of nisin and bioengineered nisin derivatives to eliminate specific pathogens and pathobionts in a predictable and beneficial manner that could steer antimicrobial regimens towards more personalized and precise medical methodologies and prevent indiscriminate microbiome damage.

We can expect that resistance development will follow any introduction of bacteriocins as new therapeutic tools. Although bioengineering programmes have already identified novel nisin variants or peptide hybrids able to overcome some resistance mechanisms, sensible stewardship, as well as limiting and tailoring applications will ensure the therapeutic success of nisin and minimize future resistance development. Despite almost a century of nisin-related research, no significant investment on the part of Pharma companies is evident in relation to the development of nisin as a drug suitable for human use. However, the fact that nisin has reached a point where it is under consideration for use as a veterinary pharmaceutical in the treatment of bovine mastitis may provide the impetus for increased investment for human clinical applications. The successful clinical development of nisin will require creative improvements in its bioavailability, stability, solubility under physiological conditions, and other parameters including pharmacokinetics and pharmacodynamics. Many knowledge gaps are still evident. Future studies will aim to provide a more complete picture of nisin biosynthesis and the membrane-associated multimeric complex, including the interactions between the transporter and protease components that may aid in the expression of a broader range of substrates. Furthermore, a better understanding of the nisin immunity mechanisms and their as yet unconfirmed co-operative nature in providing full immunity will be required in efforts to increase nisin production, as will a more in-depth analysis of the cell wall precursor target and membrane interactions with nisin to rationalize the membrane disruptive aggregation behaviour recently described.

Given the immense strides and technological improvements in nisin- and bacteriocin-related research in recent years, we have already passed a significant threshold, and it is anticipated that the tremendous potential for the development and application of tailor-made and highly specific nisin derivatives for *in vivo* antibiotic use will finally be realized at the beginning of the second century of nisin research.
